# Exploring the factors associated with diabetes and hypertension complications among refugees living in Egypt

**DOI:** 10.3389/fpubh.2026.1818580

**Published:** 2026-09-03

**Authors:** Noura Seada, Sungsoo Chun

**Affiliations:** Department of Biological, Chemical, and Global Health Science American University in Cairo, Cairo, Egypt

**Keywords:** diabetes, Egypt, hypertension, non-communicable diseases, public health, refugees, social determinants of health, MENA

## Abstract

**Introduction:**

Non-communicable diseases (NCDs) account for 74% of global deaths, with high global prevalence of diabetes and hypertension. Egypt hosts over 1 million refugees and asylum seekers, with systematic difficulties in the access to healthcare services. This study assessed factors associated with Type 2 Diabetes (T2D) and Hypertension (HTN) complications among Syrian and Sudanese refugees in Egypt, focusing on healthcare access, sociodemographic characteristics, lifestyle, and health behaviors.

**Methods:**

This cross-sectional study used a self-administered questionnaire and BMI measurement among 246 refugees diagnosed with T2D and/or HTN in Cairo, Giza, and Alexandria, recruited via convenience sampling through refugee-led organizations. The survey included standardized tools assessing diet, lifestyle, tangible social support, and access to care. Descriptive statistics and ordinal regression identified factors associated with self-reported disease complications.

**Results:**

Refugees generally showed poor health and lifestyle behaviors; unaffordability was the most reported barrier to healthcare access and medication adherence. After adjustment for sociodemographic and access-to-care variables, prolonged displacement (AOR = 1.16; 95% CI: 1.02–1.32; *p* = 0.023) and lowest tangible social support (AOR = 5.47; 95% CI: 1.26–23.67; *p* = 0.023) were associated with higher odds of hypertension complications, while not needing to seek care was associated with lower odds (AOR = 0.30; 95% CI: 0.13–0.70; *p* = 0.005). Among diabetic participants, medication non-adherence (AOR = 3.83; 95% CI: 1.01–14.53; *p* = 0.048) and failure to access needed care (AOR = 16.01; 95% CI: 3.04–84.27; *p* = 0.001) were associated with higher odds of complications, while moderate carbohydrate intake was associated with lower odds compared with high intake (AOR = 0.30; 95% CI: 0.09–0.97; *p* = 0.044). These diabetic model findings warrant cautious interpretation given the small sample and wide confidence intervals in certain variables.

**Conclusion:**

NCD complications among Syrian and Sudanese refugees in Egypt reflect a complex interplay of structural and behavioral determinants, including duration of displacement, access to care, tangible social support, medication adherence, and carbohydrate intake. These findings highlight the need for interventions addressing both clinical care and upstream social determinants of health in refugee-hosting settings.

## 1 Introduction

According to the United Nations High Commissioner for Refugees’ (UNHCR) definition, “A refugee is someone who has fled war, violence, conflict or persecution and crossed an international border to find safety in another country.” A refugee differs from a migrant, who might voluntarily relocate for various reasons (1). According to the latest reports, over 117 million people are forcibly displaced, among them are 42.5 million refugees. These numbers are bound to increase due to rising global conflicts. In this rapidly shifting scene, refugees face social injustice, human rights concerns, and disparities on various levels (2).

As of September 2025, UNHCR announced that Egypt hosts over 1 million registered refugees and asylum seekers, and the numbers are increasing; out of which 75% are Sudanese, 13% Syrians and 12% of other nationalities (3). These high numbers of refugees in Egypt are related to the background of the conflict in the neighboring countries. Starting with the Syrian conflict in 2011, which resulted in the displacement of 5 million people to neighboring countries and Europe (4). A few years later, the armed conflict erupted in Sudan on 15 April 2023, forcing large numbers of citizens to flee. By December 2023, 5.4 million people had been displaced within Sudan, and another 1.4 million had fled across the borders into the Central African Republic (CAR), Chad, Egypt, Ethiopia, and South Sudan. As a result, Egypt is now hosting the highest number of refugees in its history (5).

Refugees in Egypt live in an urban setting, predominantly in Greater Cairo, Alexandria, and Damietta (5). They often face protection concerns stemming from the complex legal situation of refugees in Egypt and the lengthy documentation process required to obtain identification documents, which are needed to access basic services (6). The health situation in Egypt is all the more challenging as the health system is undergoing a complete restructuring to establish a universal health insurance scheme for its citizens, with no clear plan yet for including refugees and migrants within this new system. Currently, out-of-pocket health expenditures account for 62% of health financing in Egypt. While the government provides subsidised healthcare services, the lack of additional resources continues to affect the quality and coverage of care imposes a substantial financial burden, especially on the most vulnerable (7). In principle, refugees have equal access to primary healthcare services as Egyptian citizens (8). However, they are not included in any social protection or insurance scheme that is currently only available to nationals (9).

Within this context, a considerable number of refugees and asylum-seekers rely on humanitarian assistance to meet their basic needs and to receive medical or psychosocial support. However, recurrent crises and emergency operations have caused donor fatigue, and most humanitarian organizations are operating with significant funding gaps (10).

This study focuses on the Noncommunicable diseases (NCDs), which are responsible for 74% of all deaths globally. Each year, 17 million people die from NCDs before age 70 (11). One in 9 people globally are suffering from Diabetes and 33% of the population worldwide between 30 and 80 years are suffering from HTN, of whom two-thirds are residing in low and middle-income countries (12–14). T2D and HTN are multifactorial diseases that require lifelong monitoring, which can affect their progression over time (15). Many studies have shown that there is a significant discrepancy in the control of T2D and HTN between migrants and host communities. These discrepancies can be attributed to pre-migration factors such as genetic predisposition and diet. They can also be attributed to post-migration lifestyle, social and psychological changes. This is in addition to financial constraints and challenges in access to healthcare services (16).

There is a slim body of research on refugees’ health in Egypt, with published studies mainly examining mental health issues (17, 18) and utilization of services (19). However, the published UN reports of 2024 point out that 17% of households reported at least one member with a chronic disease, at the top of the list were HTN (40%) and diabetes (33%) (20).

To address data scarcity and obtain comparable information that can better inform our analysis, we drew on research from neighboring countries with large refugee populations. We have also explored the prevalence of NCDs in the country of origin. Before the outbreak of war in Syria in 2011, NCDs were responsible for 77% of mortality in Syria, particularly Cardiovascular disease (CVD) (21). A cross-sectional survey of Syrian refugees in Jordan in 2015, revealed that half of all households reported at least one household member with a prior diagnosis of one of five non-communicable diseases: arthritis, CVD, chronic respiratory diseases, diabetes, or HTN (22). In 2021, a similar survey in northern Jordan also reported that 28% of household members have one or more NCDs, including Diabetes and HTN (23).

Studies in different areas in Sudan have shown a prevalence of 20% for diabetes, and 27.6% for HTN, variations exist according to location, tribe, and lifestyle factors (24, 25).

Regarding the management of the disease among refugees, studies on Syrian refugees in northern Jordan revealed that 57% reported at least one complication from HTN and diabetes. Additionally, 50% of patients aged 18 years and older did not seek care during the month before the survey, and nearly one-third missed their medications (26). In Sudan, a high prevalence of uncontrolled diabetes (85%) among Sudanese adults with T2D was reported in 2017 (27).

To better understand the factors contributing to the control of chronic diseases, we relied on the social determinants of health (SDH) framework developed by the WHO to explain the underlying factors affecting disease causation and control. This includes social, economic, and institutional mechanisms (28).

The SDH is shaped by structural mechanisms, such as gender, race, income, education, and employment, that determine the socioeconomic position of individuals, together with the social and political context. These structural determinants operate through intermediary determinants that shape health outcomes. The main categories of intermediary determinants of health are material circumstances, psychosocial factors, behavioral and/or biological factors, and the health system itself. Material circumstances include factors such as housing and neighborhood quality, as well as the financial ability to buy healthy food. Psychosocial circumstances include stressors, social support, and coping styles. Behavioral and biological factors include nutrition, physical activity, smoking, and genetic factors (29).

Reflecting on the SDH in the context of migration, gender-based structural discrimination is found to influence health needs and access to care. Women are more likely to experience different forms of violence and complications of sexual and reproductive health needs, compounded by economic vulnerability (30, 31). In addition, female-headed households are prone to experience poverty according to the health access and utilization survey (HAUS) (32).

Regarding the socioeconomic position, the vulnerability assessment of refugees in Egypt published in 2018 has shown that almost 40% of refugees live below the poverty line and a higher rate of unemployment among non-Arabic speaking refugees (33). More recently, the HAUS report published in 2024 by UNHCR has shown an alarming rate of unemployment among Sudanese refugees of 82% compared to 44% among Syrians (20). Consequently, this has extreme implications for the income levels and stability of refugee households (32).

As for educational level, evidence suggests a positive association between educational attainment and income (34), and this has been clearly demonstrated by the higher poverty rates among illiterate refugees (32). However, an increase in educational level has been reported among refugees between 2023 and 2024, reflecting a demographic change (20).

Social networks and support play an important role in an individual’s response to stress and health challenges (35). Refugees are more likely to report social isolation in destination countries than natives, and the migration process often breaks family ties (36).

Behavioral and biological factors, such as unhealthy diet and physical inactivity, contribute to around 30% of preventable morbidity and mortality from NCDs. High intake of saturated fatty acids, salt, and sugar is a risk factor for CVD, including HTN (37). Nonetheless, high carbohydrate/fat and, especially, sugar consumption contributes to obesity and the risk of insulin resistance, which affects the control of Diabetes (38).

Refugees face significant nutritional challenges, often reporting decreased food intake and limited diversity. A systematic review of dietary patterns among refugees has concluded that macro- and micronutrient intakes were affected. This results in either stunting, underweight, or anemia, particularly among children, or a high rate of overweight and obesity, associated with high blood cholesterol, increasing the risk of CVD (39).

In a regional context, a vulnerability assessment for Syrian refugees in Lebanon in 2022 showed that 57% of refugees’ households had poor or borderline food consumption. Every week, the most consumed food groups among Syrian refugees were tubers and cereals, oil/fat/butter, sugar/sweets, and, less commonly, vegetables. In contrast, fruits and meat/fish/eggs were the least consumed food groups (40).

A survey in Sudan demonstrated that sugar intake was an absolute risk factor for HTN among Sudanese individuals and The Food and Agriculture Organisation (FAO) estimated in 2010 that sugar was the second-largest source of dietary energy in the country (25).

A high prevalence of smoking among Syrian refugees in both Jordan and Canada has been reported (41, 42). Some studies linked this high smoking prevalence to the stress and mental health effects of forced displacement (43).

Physical inactivity was observed in Syrian refugees, especially among females, due to various social barriers. A cross-sectional study of Syrian and Iraqi refugees in Jordan showed that 81% of refugees lead a sedentary lifestyle (44). Similar findings were also reported on refugees in Australia and the United States, as they spend less time outdoors compared to their home country, and often lack the social support to engage in physical activity with their peers (45, 46).

This study aims to address the current research gap in non-communicable diseases among refugees. Our hypothesis is that the social determinants of health framework (SDH) can provide a valuable explanatory model for the challenges in the control of Type 2 Diabetes (T2D) and Hypertension (HTN) among refugees in Egypt.

The main objective is to assess the factors associated with self-reported complications of T2D and HTN among the refugee population in Egypt, particularly Syrian and Sudanese communities. The study will explore the effects of sociodemographic factors, diet patterns, lifestyle, social support, and health behaviors on disease control. In this study, we define disease complications as self-reported manifestations, namely diabetic foot and eye complications, among patients with T2D. In addition to renal and cardiac complications for either hypertensive, diabetic or both.

## 2 Materials and methods

### 2.1 Research design and sampling

Given the current gaps in research on non-communicable diseases among refugees in Egypt, this research contributes to the body of knowledge on this topic with a particular focus on factors contributing to the complications of HTN and T2D. The study employs a cross-sectional design and uses a self-administered questionnaire to assess socioeconomic factors, lifestyle and health behaviors, tangible social support, and access to care.

Due to challenges in approaching refugees in a diverse urban setting, we have used convenience sampling techniques to reach the target population. The sample was collected with the help of six refugee-led organizations (RLOs) in Cairo, Giza, and Alexandria.

Our case definition and inclusion criteria required that participants be registered refugees, above 30 years of age, and diagnosed with either T2D and/or HTN. Participants should preferably have been diagnosed and have been living in Egypt for at least 6 months before the survey. For verification, they were asked to bring relevant documentation of their legal status as a refugee, along with a prescription or physician’s report confirming the diagnosis and medications for hypertension, diabetes, or both. We excluded cases of type 1 diabetes, illiterate refugees, and non-Arabic speakers. Illiterate refugees were excluded to avoid interviewer effects and social desirability bias. Although non-Arabic-speaking refugees face additional and distinct barriers that are not readily generalizable to the broader refugee population and therefore warrant separate investigation, it should be noted that this subgroup represents less than 13% of the refugee population.

A sample size calculator was used to determine the minimum required sample size based on an estimated population of 800,000 registered refugees in Egypt at the time of the study. The calculation used the standard formula for estimating a population proportion, n₀ = z^2^pˆ(1−pˆ)/ε^2^, with a finite population correction applied as n = n₀/[1 + (n₀ − 1)/N]. Assuming a 90% confidence level (Z = 1.65), an expected proportion of 50%, and a margin of error of 5%, the minimum required sample size was 273, Eventually, the survey was conducted with 246 refugees based on the availability at the time of the study. We aimed to represent the nationality distribution among the refugee population in Egypt, which is predominantly Sudanese.

We explained the purpose of the research and how the data would be used for the participants before obtaining their consent to participate in the study. In addition, weight was measured to the nearest 0.1 kg using a calibrated digital scale, and height to the nearest 0.1 cm using a stature tape meter, to calculate the BMI as weight in kilograms divided by height in meters squared (kg/m^2^).

### 2.2 Survey tool

The survey instrument was developed in three adapted forms - one for participants with T2D only, one for HTN only, and one for participants with both conditions – to avoid that respondents answer to questions irrelevant to their disease state. Specific questions are included for the diabetic group: foot care, measurement of blood sugar, and diabetic foot/eye complications. Each form comprised up to 45 questions and was structured into five sections. Section 1 captured general sociodemographic information, while Section 2 addressed medical history, disease management, and complications. Section 3 examined lifestyle and behavioral factors, including dietary habits and physical activity, and Section 4 assessed access to healthcare services. Section 5 evaluated tangible social support using standardized measurement tools. All three forms were translated into Modern Standard Arabic, which is the common reading and writing language for countries in the region, to ensure accessibility across the study population.

The tool was developed based on the CAMDI (the Central America Diabetes Initiative), for diabetes, HTN and chronic disease risk factors. CAMDI’s survey is a modification of other survey tools used by the Pan American Health Organisation (PAHO) and the Centres for Disease Control and Prevention (CDC). The CAMDI tool addresses questions on diet, lifestyle, access to healthcare services, medication use, and complications (47). The section on access to healthcare was derived from questions of the HAUS tool used by UNHCR to collect yearly health data (48).

We have used the Medical Outcomes Study Social Support Survey (MOS-SS) to assess social support. And as it is a multidimensional instrument, only the tangible support subscale was administered in this study (49). Internal consistency of the four tangible support items was good, with Cronbach’s α = 0.867 (*n* = 231).

The questions were modified to fit the context of refugees and their cultural and socioeconomic status as follows; the question on physical activity was formulated to ask about activities refugees can easily engage in such as walking or using the stairs. Similarly, questions on diet were designed to capture diet staples available in the Egyptian market, and the wording for the different food groups was checked in the survey pilot.

The survey was piloted with 10 community volunteers (Syrian and Sudanese) of both genders to assess whether there were any difficulties with the language or the formulation of the questions. The duration of the survey was observed, and participants were asked for feedback on the questions, including whether they experienced any difficulties understanding them or felt that any were sensitive or intrusive. After the evaluation of all participants’ observations, a question on how they rated their income was removed to avoid confusion or sensitivities related to sharing income level. We used the help of volunteers from the 6 RLOs to assist with data collection, and vouchers from a commercial food retailer were provided to encourage participation.

### 2.3 Ethical considerations

The Institutional Review Board (IRB) at the American University in Cairo (AUC) reviewed the study proposal and concluded that the data subjects face minimal risk on September 24th, 2024. Data collection began in February 2025 and concluded in July 2025, lasting 6 months.

The survey form was distributed to participants in person, with the consent form attached. We thoroughly explained the study’s purpose and the use of the data to the candidate participants. Participation in the survey was voluntary, and each participant signed an informed consent.

### 2.4 Data analysis

The data were recorded in Microsoft Excel for Microsoft 365. After editing and logical checking, they were analyzed using SPSS version 26.0. We performed data wrangling to group continuous data, such as age, years in Egypt, and BMI, into categories for bivariate analysis. Age was dichotomized into two categories (above and below 50 years), and duration of stay was divided in a similar manner into less than and more than 4 years, guided by the mean and Standard Deviation (SD). Similarly, BMI scores were grouped according to the World Health Organisation (WHO) criteria into Normal, Overweight and Obese (50). However, continuous data were used in their original form in the regression model. For certain questions, responses of “Not sure” and “I do not know” were treated as negative responses and combined with “No” to simplify analysis. In addition, responses to social support questions were used to compute the tangible social sum and tangible social mean scores for easier interpretation, and inclusion within the regression models.

Descriptive analyses, such as frequency, percentage, mean, and SD, were performed as appropriate for categorical and continuous variables. The chi-square test was used to assess the significance of the relationship between the categorical variables: demographic factors on the one hand and the categorical disease state and complications, BMI, access to care, and utilization of health services.

In addition, we used the Independent-samples t-test and one-way ANOVA to determine mean differences in age, years in Egypt, BMI, years of smoking, age at first diagnosis, and the tangible social mean across the different demographic variables. We have set the α level at 0.05 (*p*-value) and a 95% confidence interval to assess the distribution in the population. To further test the hypothesis, a correlation analysis was performed on continuous variables, and an ordinal regression analysis was done to examine the relationships among access to healthcare, sociodemographic variables, and disease complications.

Bivariate comparisons (chi-square tests, independent-samples *t*-tests, and one-way ANOVA) were used descriptively to explore associations between sociodemographic characteristics and disease-related variables and should be interpreted as exploratory given the number of comparisons performed; and are not used to draw confirmatory conclusions. For chi-square analyses, Fisher’s exact test was used in place of the chi-square approximation where expected cell counts fell below 5 (see Supplementary material). Normality of continuous variables was not formally tested; given the sample sizes involved, *t*-tests and ANOVA were considered reasonably robust to moderate deviations from normality, and this is acknowledged as a limitation.

Ordinal regression analysis was performed with two models, one for the diabetic complications, selectively for the diabetic group, and a model for renal and cardiac complications run on the whole sample. Outcomes are self-reported disease complications, which serve as a proxy for poor control. While the independent variables are sociodemographic, behavioral, lifestyle factors, as well as access to care, medication adherence, and tangible social support.

For this analysis, access to care and medication compliance variables were each derived from two sequential survey questions. For access to care, participants were first asked whether they had needed to receive care or treatment in a health facility during the preceding 3 months; those who responded affirmatively were then asked whether they were able to access that care. These two responses were combined into a single three-category variable: “did not seek care” (did not need care coded = 0), “sought care but failed to access it” (needed care but reported being unable to access it, coded = 1), and “accessed care” (needed and successfully accessed care, coded = 2).

Medication compliance was constructed analogously from two sequential questions: whether participants were taking their medications regularly, and, if so, whether they were taking the exact medication type and dosage prescribed by their physician. Responses were combined into three categories: “not compliant” (not taking medications regularly, coded = 0), “Regular, not exact” (taking medications regularly but not exact prescription, coded = 1) and “Exact prescription” (coded = 2).

## 3 Results

### 3.1 Sample characteristics and descriptive analysis

#### 3.1.1 Sociodemographic characteristics

The sample was predominantly Sudanese (84.4%), with Syrians comprising 15.6%, and females accounting for 73.6% of participants. Educational attainment was relatively high, with 36.8% having completed high school and 35.1% having attained university-level education; however, 76.1% of participants reported being unemployed at the time of the study (Table 1).

**Table 1:** Sociodemographic characteristics of the sample.

Variable	Category	*N* (%)
Nationality	Syrian	38 (15.6)
	Sudanese	206 (84.4)
Gender	Female	181 (73.6)
	Male	65 (26.4)
Educational level	None	8 (3.3)
	Elementary	48 (19.8)
	High school	89 (36.8)
	Vocational training	12 (5.0)
	University	85 (35.1)
Work	Yes	57 (23.9)
	No	181 (76.1)
Age (years)	Mean (SD)	53.07 (11.31)
Years in Egypt	Mean (SD)	3.58 (4.36)

N(%), number and percentage, SD, standard deviation. Percentages are calculated on the number of valid responses to each item; denominators therefore vary between items because of item-level missing data.

The mean age of participants is (53 ± 11.31) years, in line with our sampling strategy targeting people diagnosed with HTN and/or T2D, who are mostly above 30 years. The average reported duration of stay is (3.5 ± 4.36) years. The mean duration of stay in Egypt was longer among Syrians (11.2 ± 5.8 years) than among Sudanese [2.3 ± 2.2 years; *t*(35.8) = 8.95, *p* < 0.001], reflecting the fact that the Syrian crisis preceded the Sudanese war by a decade (Table 1).

#### 3.1.2 Medical history and health behaviors

The majority of participants reported having HTN (70.7%), followed by T2D (61.8%), with 38.2% reporting both conditions (Table 2). T2D was more prevalent among males than females (72.3% vs. 58.0%; *p* = 0.042), while HTN was significantly more common among Syrians than Sudanese (86.8% vs. 67.5%; χ^2^ = 5.79, *p* = 0.016) (Supplementary Table S1).

**Table 2:** Medical history.

**Variable**	**Category**	**N (%)**
HTN	Yes	174 (70.7)
	No	72 (29.3)
T2D	Yes	152 (61.8)
	No	94 (38.2)
Combined (HTN + T2D)	Yes	94 (38.2)
Hypertensive participants on medications	Yes	154 (92.8)
	No	12 (7.2)
Diabetic participants on medications	Yes	141 (95.3)
	No	7 (4.7)
Type of diabetes medication	Insulin injections and pills	39 (26.0)
	Pills only	91 (60.7)
	Insulin only	14 (9.3)
	None	6 (4.0)
Family history of HTN	Yes	148 (87.1%)
	No	21 (12.4)
	I do not know	1 (0.6)
Family history of T2D	Yes	117 (79.1)
	No	25 (16.9)
	I do not know	6 (4.1)
BMI (kg/m^2^)	Mean (SD)	29.49 (5.88)
Age at HTN diagnosis (years)	Mean (SD)	42.36 (11.06)
Age at T2D diagnosis (years)	Mean (SD)	43.24 (11.72)

N(%), number and percentage; HTN, hypertension; T2D, type 2 diabetes; BMI, body mass index; SD, standard deviation. Percentages are calculated on the number of valid responses to each item; denominators therefore vary between items because of item-level missing data.

The dual burden of T2D and HTN was strongly associated with employment status, with non-working participants showing markedly higher rates than working participants (38.7% vs. 12.3%; *p* < 0.001) (Supplementary Table S1).

Approximately 80% of both diabetic and hypertensive participants reported a positive family history of their respective condition; however, as the survey did not specify degree of kinship, a genetic association cannot be reliably inferred from this finding.

The mean BMI is 29.4, at the upper limit of the overweight range (Table 2) and was significantly higher in females than males (30.2 ± 6.0 vs. 27.4 ± 4.9 kg/m^2^; *p* < 0.001) (Supplementary Table S3).

Regarding health behaviors, participants were asked about the frequency of blood pressure and blood sugar measurement and more than half of the participants reported measuring only occasionally or when they felt they needed to (Table 3). Among diabetic patients, (74.1%) had not noticed any signs in their feet in the last 2 weeks, and the majority (33.9%) did not habitually inspect their feet for these signs. While responses wearing suitable shoes’ size exhibited a mixed pattern (Table 3).

**Table 3:** Lifestyle and health behaviors.

Variable	Category	*N* (%)
Frequency of blood pressure measurement	Weekly	22 (13.1)
	Monthly	47 (28.0)
	Occasionally / when needed	94 (56.0)
	I do not measure	5 (3.0)
Frequency of blood sugar measurement	Daily	16 (10.7)
	Weekly	23 (15.4)
	Monthly	53 (35.6)
	When I feel I need to	57 (38.3)
Noticed blisters/cuts/swelling in feet in the past 2 weeks	No	109 (74.1)
	Yes	38 (25.9)
Habit of checking feet for these signs	Yes, all the time	40 (28.8)
	Sometimes	33 (23.7)
	Rarely	13 (9.4)
	No	47 (33.8)
	I do not know	6 (4.3)
Suitable footwear (indoor and outdoor)	Yes, all the time	55 (36.7)
	Sometimes	25 (16.7)
	Rarely	12 (8.0)
	No	55 (35.7)
	I do not know	3 (2.0)
Current tobacco smoking	Daily	18 (7.4)
	Less than daily	5 (2.1)
	Not at all	217 (89.7)
	I do not know	2 (0.8)
Years since starting daily smoking	Mean (SD)	25.50 (15.43)
Physical activity, ≥30 min (last 7 days)	4 times or more	74 (30.7)
	3 times	39 (16.2)
	2 times	64 (26.6)
	≤1 time	64 (26.6)
Fast food / food not prepared at home (times/week)	4 times or more	2 (0.9)
	3 times	14 (6.3)
	2 times	24 (10.7)
	≤1 time	184 (82.1)
Meat/chicken (times/week)	4 times or more	30 (12.7)
	3 times	37 (15.6)
	2 times	54 (22.8)
	≤1 time	116 (48.9)
Fish (times/week)	4 times or more	1 (0.4)
	3 times	4 (1.7)
	2 times	17 (7.2)
	≤1 time	214 (90.7)
Fruit (servings/day)	2 or more	21 (8.9)
	1 to 2	56 (23.7)
	≤1	99 (41.9)
	Almost none	60 (25.4)
Vegetables (servings/day)	2 or more	67 (27.8)
	1 to 2	113 (46.9)
	≤1	49 (20.3)
	Almost none	12 (5.0)
Rice/pasta/bread (servings/day)	4 or more	43 (17.8)
	3	68 (28.1)
	2	77 (31.8)
	≤1	54 (22.3)
Oil/fat used for cooking	Vegetable oil	185 (76.4)
	Natural butter	11 (4.5)
	Vegetable margarine	5 (2.1)
	Other	1 (0.4)
	Mixture	40 (16.5)

N(%), number and percentage. Percentages are calculated on the number of valid responses to each item; denominators therefore vary between items because of item-level missing data.

Most participants relied on home-prepared food rather than fast food (82.1%), but overall dietary patterns were poor, characterized by high carbohydrate intake alongside low consumption of meat, chicken, fish, vegetables, and fruit (Table 3).

Nearly 90% of our sample are non-smokers, among those who reported smoking regularly, the mean duration of 25.50 ± 15.43 years (Table 3). On tangible social support, the majority highly rated their perception of the support they received from their family and caregivers (Table 4). Males reported higher perceived support than females (3.71 ± 1.13 vs. 3.17 ± 1.23; *p* = 0.002) (Supplementary Table S3). While the scores were considerably lower among Syrians than among Sudanese (2.85 ± 1.22 vs. 3.39 ± 1.22; *p* = 0.02) (Supplementary Table S3).

**Table 4:** Tangible social support.

Variable	Category	*N* (%)
Someone to help when confined to bed	None of the time	48 (20.3)
	A little of the time	42 (17.7)
	Some of the time	49 (20.7)
	Most of the time	48 (20.3)
	All of the time	50 (21.1)
Someone to take you to the doctor if needed	None of the time	31 (13.0)
	A little of the time	34 (14.2)
	Some of the time	45 (18.8)
	Most of the time	55 (23.0)
	All of the time	74 (31.0)
Someone to prepare meals if unable to do so yourself	None of the time	48 (19.9)
	A little of the time	31 (12.9)
	Some of the time	32 (13.3)
	Most of the time	55 (22.8)
	All of the time	75 (31.1)
Someone to help with daily chores if sick	None of the time	35 (14.5)
	A little of the time	39 (16.2)
	Some of the time	43 (17.8)
	Most of the time	46 (19.1)
	All of the time	78 (32.4)

N(%), number and percentage. Percentages are calculated on the number of valid responses to each item; denominators therefore vary between items because of item-level missing data.

Age was also positively correlated with tangible social support sum score (r = 0.288, *p* < 0.01), suggesting that older respondents reported stronger social networks and greater perceived support (Supplementary Table S4).

#### 3.1.3 Access to care and disease outcomes

Most respondents reported that a healthcare professional provided them with information about the treatment plan (75%) and were taking their medications regularly (85%). However, around 40% were not taking the exact prescribed regimen; commonly citing financial constraints (Table 5).

**Table 5:** Access to care and disease complications.

Variable	Category	*N* (%)
Healthcare professional explained treatment plan and complications	No	57 (24.1)
	Yes	180 (75.9)
Taking medications regularly	No	32 (15.0)
	Yes	181 (85.0)
Taking exact medication type/dosage prescribed	No	93 (39.6)
	Yes	142 (60.4)
If no, reason	Financial reasons	60 (66.7)
	Unavailability of medications	20 (22.2)
	Too many medications	7 (7.8)
	Inability to organize intake	7 (7.8)
Medications changed due to complications	Changed partially	54 (23.9)
	Changed totally	30 (13.3)
	Change in dosage	44 (19.5)
	Nothing	98 (43.4)
Needed care in a health facility (last 3 months)	Yes	126 (51.9)
	No	107 (44.0)
	I do not know	10 (4.1)
Was able to access care	No	56 (33.3)
	Yes	116 (66.7)
If yes, where care was received	Private hospital/clinic	29 (21.0)
	Public hospital	52 (37.7)
	NGO/charity clinic	48 (34.8)
	Other	9 (6.5)
If no, reason	Entity refused to provide care	2 (2.0)
	Could not afford user fees	51 (51.5)
	Too far / transportation issues	12 (12.2)
	Could not get time off work	6 (6.1)
	Did not know where to go	8 (8.1)
	Did not like health services/staff	1 (1.0)
	Language barriers	4 (4.0)
	Lack of identity documents	3 (3.0)
	Other	12 (12.1)
	I do not know	5 (5.1)
Condition under control with medications	Yes, totally	88 (36.2)
	Relatively	112 (46.1)
	No	12 (4.9)
	I do not know	31 (12.8)
Diabetic foot complications in the last year	Yes	17 (11.6)
	No	129 (87.8)
	I do not know	1 (0.7)
Diabetic eye complications in the last year	Yes	86 (58.1)
	No	53 (35.8)
	I do not know	9 (6.1)
Renal function affected (doctor-reported)	Yes	34 (14.2)
	No	180 (75.3)
	I do not know	25 (10.5)
Heart attack, stroke, or related hospital admission	Yes	36 (15.0)
	No	195 (81.3)
	I do not know	9 (3.8)

N(%), number and percentage. Percentages are calculated on the number of valid responses to each item; denominators therefore vary between items because of item-level missing data. Reasons for not taking the exact prescribed medication were a multiple-response item. The access-to-care items are reported here as recorded: some participants answered the follow-up question on whether care was obtained despite having indicated that they did not need care, so the number of responses to that item exceeds the number reporting a need for care. These off-protocol responses were reassigned when the derived access-to-care variable used in the regression models was constructed.

Older participants were significantly more likely to take their medications regularly than younger participants (89.1% vs. 77.5%, χ^2^ = 5.06, *p* = 0.025). A striking age-related difference was also observed in the perception of disease control (χ^2^ = 15.515, *p* < 0.001), with older adults more likely to report that their condition was “totally under control” (43.4% vs. 22.6%), whereas younger adults more often stated that their condition was “not under control” (28.0% vs. 11.7%) (Supplementary Table S1).

Syrians were less likely to adhere to exact prescribed medications compared to Sudanese (34.2% vs. 65.6%, *p* < 0.001) and Sudanese reported greater perceived control over their health (38.4% vs. 23.7%; *p* = 0.028). It was notable as well that adherence to the exact prescribed dosage declined with longer residence χ^2^ = 9.99, *p* = 0.002) (Supplementary Table S1).

Just over half of respondents (51.9%) have sought healthcare services in the last 3 months and were able to access them for the most part (67%), largely relying on public health facilities and NGO/charity clinics. Among those who failed to receive care, the main barrier mentioned was the unaffordability of services (51.5%), followed by distance and transportation challenges (12.1%) (Table 5).

Among diabetic participants, approximately 11% reported diabetic foot complications and 58% reported diabetic eye complications during the preceding year. Across all sample, 14–15% reported experiencing renal or cardiac complications during the same period (Table 5).

### 3.2 Ordinal regression

The results of the ordinal regression analyses are presented below. Independent variables were selected on the basis of both statistical and theoretical relevance. Gender, nationality, tangible social support, duration of stay, and BMI were included in both models given their significant associations in the bivariate analysis, alongside medication adherence and access to care to capture the influence of health system barriers. For the diabetes model, carbohydrate intake was additionally incorporated as a dietary indicator, given its well-documented effect on glycemic control in literature. For the cardiac and renal complications model, age was included on due to its significant bivariate association with these outcomes.

Given the relatively small number of valid cases available for regression analysis, variable selection was deliberately restricted to those with the strongest statistical or theoretical justification. This approach was intended to capture the most relevant associations, while avoiding model overcrowding and the risk of overfitting.

We conducted an ordinal regression analysis of foot and eye complications for participants reportedly diagnosed with T2D, while the analysis for cardiac and renal complications was extended for the whole sample of both Diabetic and hypertensive participants. The outcome variable comprised three ordered categories (no complications, one complication, and two or more complications).

A comparison of included and excluded participants across all variables selected for analysis revealed no statistically significant differences, with the exception of medication adherence among the diabetes-eligible subgroup (*p* = 0.025). Excluded participants were more likely to report taking their medications regularly but not exactly as prescribed (52.0% vs. 24.5%), and less likely to report taking the exact prescription (40.0% vs. 63.6%), compared with included participants; the proportion reporting non-compliance was similar between groups (8.0% vs. 11.8%). Since medication compliance emerged as a significant predictor of diabetic complications in the final model, this difference should be acknowledged as a potential source of complete-case bias (Supplementary Table S8).

Multicollinearity among variables was assessed using variance inflation factors (VIF) and tolerance statistics, using linear regression models specified with the same variable sets as the ordinal regression models. All VIF values were well below the conventional threshold of 5 for both the diabetes model (range: 1.02–2.11) and the cardiac/renal complications model (range: 1.04–2.42) (Supplementary Table S9). The highest VIF values in both models were observed for nationality and years in Egypt, reflecting the expected correlation between these variables given the markedly longer duration of residence among Syrian compared with Sudanese participants in this sample; this correlation was not severe enough to compromise the stability of coefficient estimates.

#### 3.2.1 Cardiac and renal complications

The ordinal logistic regression model examining factors associated with cardiac and renal complications included 169 participants with complete data on all covariates.

After adjustment for all covariates, three variables emerged as statistically significant associations to complications. Longer duration of residence in Egypt was associated with higher odds of complications (AOR = 1.16; 95% CI: 1.02–1.32; *p* = 0.023), indicating that each additional year of displacement was associated with a 16% increase in the odds of greater complication burden, independent of age. Participants who reported not needing to seek care in the preceding 3 months had significantly lower odds of reporting complications (AOR = 0.30; 95% CI: 0.13–0.70; *p* = 0.005), which may be explained by a severity-driven pattern of healthcare utilization. Participants reporting the lowest level of tangible social support had markedly higher odds of complications compared with those reporting the highest support level (AOR = 5.47; 95% CI: 1.26–23.67; *p* = 0.023), indicating a pronounced social gradient in HTN-related outcomes (Table 6).

**Table 6:** Ordinal logistic regression of factors associated with renal and cardiac complications (*n* = 169).

Variable	Category	AOR	95% CI	*p*-value
Gender	*Male (ref: Female)*	1.04	0.39–2.78	0.938
Nationality	*Sudanese (ref: Syrian)*	1.78	0.39–8.23	0.459
Years in Egypt (per year)	*—*	**1.16**	**1.02–1.32**	**0.023**
BMI (kg/m^2^)	*—*	0.99	0.92–1.06	0.752
Age (per year)	*—*	1.04	1.00–1.09	0.056
Medication compliance	*Not compliant (ref: Exact prescription)*	1.61	0.53–4.86	0.400
	*Regular, not exact (ref: Exact prescription)*	0.82	0.30–2.26	0.698
Healthcare access	*Did not seek care (ref: Accessed care)*	**0.30**	**0.13–0.70**	**0.005**
	*Sought but failed to access (ref: Accessed care)*	1.03	0.26–4.10	0.965
Tangible social support score	*1.00 – lowest (ref: 4.00 – highest)*	**5.47**	**1.26–23.67**	**0.023**
	*2.00 (ref: 4.00)*	0.58	0.17–1.89	0.362
	*3.00 (ref: 4.00)*	1.63	0.62–4.29	0.323

AOR, adjusted odds ratio; CI, confidence interval; BMI, body mass index. Outcome: ordered categories of HTN-related complication severity (no complications, one complication, two or more complications). Model fit: −2 log-likelihood χ^2^(12) = 40.21, *p* < 0.001; Nagelkerke *R*^2^ = 0.283; test of parallel lines χ^2^(12) = 9.44, *p* = 0.665. Bold values indicate statistically significant associations (*p* < 0.05).

The model demonstrated a significant improvement over the intercept-only model [χ^2^(12) = 40.21, *p* < 0.001; Nagelkerke *R*^2^ = 0.283], and the proportional odds assumption was upheld [test of parallel lines: χ^2^(12) = 9.44, *p* = 0.665], supporting the appropriateness of the ordinal approach.

#### 3.2.2 Diabetic foot and eye complications

The ordinal logistic regression model examining factors associated with diabetic foot and eye complications included 110 participants with complete data on all covariates.

After adjustment for all covariates, healthcare-related factors emerged as the strongest associations with disease complications. Participants who sought care but were unable to access it had substantially higher odds of complications compared with those who successfully accessed care (AOR = 16.01; 95% CI: 3.04–84.27; *p* = 0.001), and medication non-compliance was associated with significantly higher odds of complications relative to taking the exact prescription (AOR = 3.83; 95% CI: 1.01–14.53; *p* = 0.048). With respect to diet, consumption of 2 servings/day of rice/pasta/bread showed a significant association with lower odds of complications compared to consuming 4 or more servings/day (AOR = 0.30; 95% CI: 0.09–0.97; *p* = 0.044) (Table 7).

**Table 7:** Ordinal logistic regression of factors associated with diabetes-related complications (*n* = 110).

Variable	Category	AOR	95% CI	*p*-value
Gender	*Male (ref: Female)*	0.43	0.16–1.12	0.083
Nationality	*Sudanese (ref: Syrian)*	0.59	0.11–3.05	0.527
Years in Egypt (per year)	*—*	1.09	0.94–1.27	0.261
BMI (kg/m^2^)	*—*	1.00	0.93–1.07	0.952
Rice/pasta/bread (servings/day)	*≤1 (ref: 4+)*	0.92	0.25–3.40	0.900
	*2 (ref: 4+)*	**0.30**	**0.09–0.97**	**0.044**
	*3 (ref: 4+)*	1.27	0.43–3.74	0.660
Medication compliance	*Not compliant (ref: Exact prescription)*	**3.83**	**1.01–14.53**	**0.048**
	*Regular, not exact (ref: Exact prescription)*	0.51	0.19–1.41	0.194
Healthcare access	*Did not seek care (ref: Accessed care)*	1.25	0.54–2.88	0.606
	*Sought but failed to access (ref: Accessed care)*	**16.01**	**3.04–84.27**	**0.001**
Tangible social support score	*1.00 – lowest (ref: 4.00 – highest)*	1.75	0.28–10.91	0.546
	*2.00 (ref: 4.00)*	0.88	0.31–2.55	0.819
	*3.00 (ref: 4.00)*	0.82	0.29–2.27	0.697

AOR, adjusted odds ratio; CI, confidence interval; BMI, body mass index. Outcome: ordered categories of DM-related complication severity (no complications, one complication, two or more complications). Model fit: −2 log-likelihood χ^2^(14) = 30.00, *p* = 0.008; Nagelkerke *R*^2^ = 0.283; test of parallel lines χ^2^(14) = 19.24, *p* = 0.156. Bold values indicate statistically significant associations (*p* < 0.05).

The model demonstrated a significant improvement over the intercept-only model [χ^2^(14) = 30.00, *p* = 0.008; Nagelkerke *R*^2^ = 0.283], and the proportional odds assumption was upheld [test of parallel lines: χ^2^(14) = 19.24, *p* = 0.156], supporting the appropriateness of the ordinal approach.

## 4 Discussion

There is a perceivable gap in research on healthcare access and outcomes among refugees in Egypt, which is one of the main drivers of our research topic.

This paper explores the factors associated with self-reported complications of T2D and HTN among Syrian and Sudanese refugees residing in Egypt, with a particular focus on healthcare access, sociodemographic characteristics, lifestyle factors, and health behaviors. The findings suggest that controlling NCDs in this population was profoundly influenced by structural barriers related to displacement and intermediary determinants such as duration of displacement and access to care, aligning with the SDH framework.

The main variables emerging in relation to disease complications in our analysis of this sample of the refugee population in Egypt are duration of displacement, with higher odds of complications for each additional year. Decreased tangible social support and inability to access care were also found to be significant associations to disease complications. Specifically for diabetic foot and eye complications, adherence to medications and carbohydrate consumption were the main associations with complications among the diabetic participants.

We used the SDH framework to demonstrate our descriptive results and analysis in sections below.

### 4.1 Sociodemographic position

Older participants reported a more positive perception of their disease control and were more likely to take their medications regularly than younger participants, this contrasts with the general evidence that older patients face greater challenges in medication adherence (51, 52). One possible explanation is that older adults, who are more often unemployed (mean age of employment 48.1 ± 10.44) (Supplementary Table S3), may experience greater stability or motivation for self-management, whereas younger, working individuals may face more competing demands.

Notable differences as well was observed in nationality, with Syrian participants reported a higher rate of HTN, compared to Sudanese, and accordingly experienced higher rates of serious complications, including renal impairment (35.1% vs. 10.5%; *p* < 0.001) and prior heart attack or stroke (37.8% vs. 10.9%; *p* < 0.001) (Supplementary Table S1).

Gender differences were pronounced across several variables. Females showed significantly higher rates of obesity (48.2%) and a higher mean BMI (30.2 kg/m^2^) than males (Supplementary Table S3), consistent with data from other refugee-hosting countries (53, 54).

Long-term residents reported significantly diminished perceived control over their health conditions, and adherence to prescribed medication dosages declined markedly with prolonged stay (56.4% vs. 32.6%, *p* = 0.002). Moreover, longer duration of displacement was significantly associated with higher rates of both renal and cardiac complications (33.9% vs. 7.7%, *p* < 0.001) and (32.1% vs. 9.9%, *p* < 0.001), respectively (Supplementary Table S1). Even with controlling for age, ordinal regression analysis confirmed that each additional year of residence was independently associated with increased odds of cardiac and renal complications (AOR = 1.16; 95% CI: 1.02–1.32; *p* = 0.023) (Table 6). These findings are consistent with reports from the WHO on refugee health across various destination countries, which similarly document a decline in health status with prolonged displacement. This progressive deterioration likely reflects the compounding challenges refugees encounter in sustaining lifelong treatment regimens and securing consistent medical follow-up, both of which are critical to the effective management of non-communicable diseases (NCDs) (55, 56).

Unemployment is a well-established social determinant of health, with a robust body of literature demonstrating that unemployed individuals are at significantly greater risk of carrying more than one chronic disease (57). The present data are consistent with this evidence, as unemployed participants exhibited a substantially higher prevalence of the dual burden of disease compared to their employed counterparts (38.7% vs. 12.3%, *p* < 0.001) (Supplementary Table S1). Refugees face limited access to the formal job market in Egypt due to the complex legal structure and documentation requirements, which in turn constrains their capacity to afford healthcare (58).

### 4.2 Tangible social support

A noteworthy finding is the comparatively lower mean tangible social score observed among Syrian participants relative to their Sudanese counterparts (Supplementary Table S3). This disparity may be partly explained by proxy indicators suggesting that Sudanese households tend to be larger, with women bearing an average of five to six children over their reproductive lifetime (59, 60).

Males scored higher on the tangible social support mean scores compared to females in our sample, echoing a broader pattern among refugee populations in which women report greater social isolation in countries of asylum (36).

Additionally, old age was positively correlated with the tangible social mean score (Supplementary Table S4). However, this should be interpreted with caution, since older patients were also more likely to report a positive perception of disease control (Supplementary Table S1). We should consider acquiescence bias, which is common among this age group (61), and the general evidence pointing to poor health control perception among the older adults (62).

Ordinal regression demonstrated that participants reporting the lowest level of tangible social support had markedly higher odds of more severe complications than those reporting the highest level (AOR = 5.47; 95% CI: 1.26–23.67; *p* = 0.023) (Table 6), reinforcing the role of social support as a determinant of health outcomes in this population.

### 4.3 Behavioral and lifestyle factors

The sample’s mean BMI of 29.4 places the population at the upper limit of the overweight range (Table 2), which aligns with existing regional data reporting high rates of overweight and obesity, especially among females (63).

Although most participants relied predominantly on home-prepared food, dietary patterns were characterized by high consumption of staples such as rice/pasta/bread and low consumption of meat, fruit, and vegetables (Table 3). This poor nutritional pattern is a known risk factor for cardiovascular diseases and insulin resistance (38), consistent with literature describing the limited availability of nutritionally adequate food in the low-resource settings where many refugees reside (39).

Observing the effects on health outcomes, our analysis shows that diabetic participants reporting moderate carbohydrate intake (2 servings/day of rice, pasta, or bread) had significantly lower odds of more severe complications than those reporting the highest intake (≥4 servings/day) (AOR = 0.30; 95% CI: 0.09–0.97; *p* = 0.044) (Table 7), underscoring the potential influence of this particular food group on disease control.

Most participants with T2D did not report proper foot care practices; a small percentage reported regularly inspecting their feet. Furthermore, most participants did not regularly measure their blood pressure/sugar, but only when they needed to (Table 3), reflecting poor health education and behaviors.

Medication non-compliance especially for diabetic participants, emerged as a significant association with complications after adjustment for other factors (AOR = 3.83; 95% CI: 1.01–14.53; *p* = 0.048), underscoring the central role of treatment adherence in preventing disease progression (Table 7).

The low smoking rate in our sample (10%) might be attributed to the predominance of females in the sample (Table 3). Smoking is generally more common among males, given that they have more access to smoking in public spaces (64).

### 4.4 Access to care and health outcomes

Although most respondents confirmed that they are taking their medications regularly (85%). However, around 40% are not taking the exact prescription; the most frequently reported reason was financial constraints (Table 5).

Half of respondents (51.9%) have sought healthcare services in the last 3 months and were able to access them for the most part (67%). Those who sought care relied more on public health facilities and NGO/charity clinics. Those who failed to receive care reported that the main barrier was the unaffordability of services (51.5%), reflecting the economic burden of healthcare services (Table 5).

More importantly, two distinct patterns emerged across the regression models. Among participants with HTN, those who reported not needing to seek care in the preceding 3 months had significantly lower odds of renal and cardiac complications than those who had accessed care (AOR = 0.30; 95% CI: 0.13–0.70; *p* = 0.005) (Table 6). Conversely, among diabetic participants, those who sought care but were unable to access it had substantially higher odds of complications than those who successfully accessed care (AOR = 16.01; 95% CI: 3.04–84.27; *p* = 0.001) (Table 7). A possible interpretation is that health services utilization might be driven by severity of complications: participants with milder or better-controlled disease may be less likely to require care in the first place, while those who do develop complications are more likely to seek care despite facing barriers to actually accessing it. Further investigation is needed to better understand the health-seeking behavior of refugees in this context, as financial constraints and unfamiliarity with the host country’s health system may all contribute to this pattern.

### 4.5 Limitations

Several methodological limitations should be considered when interpreting the findings of this study. First, the cross-sectional design precludes causal inference; although significant associations were observed between prolonged displacement and adverse health outcomes, the temporal sequence of these events cannot be definitively established. To partially address this limitation, questions on complications were restricted to the year preceding the survey; however, this approach mitigates rather than eliminates the underlying ambiguity.

Second, the use of convenience sampling, necessitated by the practical difficulty of reaching a dispersed urban refugee population, introduces the potential for selection bias. The sample is therefore unlikely to be representative of the broader refugee population in Egypt, particularly individuals not affiliated with refugee-led organizations or residing outside major urban centers. Relatedly, the exclusion of illiterate participants and non-Arabic speakers, while methodologically necessary to minimize interviewer effects and language-related measurement error, further limits the generalizability to this particular group. Further studies, preferably qualitative, should investigate more closely this segment of the refugee population.

Third, reliance on a self-administered questionnaire means that data on disease history, medication adherence, dietary patterns, and perceived disease control are inherently self-reported and susceptible to recall and social desirability bias. As several challenges in the field render it difficult to obtain clinical data supporting the control of the disease, such as HbA1c level or retinal eye exams, we relied on self-reported symptoms of complications. This approach is subject to misclassification arising from variation in participants’ health literacy and understanding of symptoms.

Fourth, the demographic composition of the sample exhibited notable imbalances by nationality and gender. The predominance of Sudanese participants (84.4%) was an intentional design choice, reflecting current demographic estimates of the refugee population in Egypt (3); however, we were not able to balance gender representation in our data collection as it relied on participants choice and availability, which may limit comparative findings across both nationality and gender subgroups. Moreover, nationality related differences especially relating to the rate of complications might be confounded by age, or duration of displacement which is notably longer for Syrians.

Fifth, although the ordinal regression model has pointed to interesting findings when it comes to the variables associated with the disease complications. The diabetes model in particular retains a relatively modest sample size given the number of model parameters, and several adjusted odds ratios showing wide confidence intervals; notably for medication compliance and access to care. These wide intervals primarily reflect genuine sparsity within specific variables rather than instability introduced by the variable correction or multicollinearity among predictors. In addition, significant differences in medication adherence patterns were observed between included and excluded cases in the diabetes model, contributing to a potential selection bias. Given these constraints, findings from regression analysis, particularly the diabetes model, should be regarded as exploratory associations, and point estimates with wide confidence intervals are to be interpreted with appropriate caution.

Finally, the absence of a non-displaced comparison group, whether drawn from the host population or from a non-refugee community of similar nationality, limits our ability to determine whether the associations observed are specific to the experience of displacement or instead reflect broader regional patterns in NCD risk and management.

## 5 Conclusion and recommendations

This study examined factors associated with self-reported complication burden among Syrian and Sudanese refugees living with T2D and/or hypertension in Egypt, within the framework of the social determinants of health. The findings suggest that self-reported NCD complications in this population are associated not solely with clinical or behavioral factors, but with a broader set of structural and intermediary determinants linked to the experience of displacement.

Duration of residence in Egypt showed a notable association with complication burden, with each additional year independently linked to higher odds of self-reported cardiac and renal complications. This pattern is consistent with a possible cumulative effect of prolonged displacement on health, although the cross-sectional design precludes drawing causal conclusions. Limited access to care and lower tangible social support were similarly associated with a higher complication burden, pointing to the potential relevance of healthcare availability and social networks to disease outcomes in this population. At the behavioral level, medication non-adherence and higher carbohydrate consumption were associated with greater complication burden, particularly among participants with T2D.

Sociodemographic factors were also associated with the health profile of this population. Unemployment was linked to a higher burden of co-occurring chronic disease, while gender differences were observed in both obesity prevalence and social isolation, with women appearing more affected in both respects. Notably, despite relatively high educational attainment in the sample, education was not associated with improved disease outcomes, suggesting that structural barriers to care may outweigh the potential benefits of education in this context.

These findings may have implications for policymakers, healthcare providers, and humanitarian organizations operating in refugee-hosting countries. Interventions targeting refugee health may benefit from extending beyond clinical management to consider upstream determinants of health, including employment, financial barriers to care, and social integration. As this study relied on a cross-sectional design and self-reported outcomes among a non-random sample, longitudinal and representative studies are needed to clarify the temporal and potentially causal pathways linking displacement and NCD outcomes, and to inform evidence-based policies to support the health of refugee populations over time.

## Data Availability

The datasets presented in this article are not readily available, given the sensitivity of health and identity-related information in this vulnerable population, de-identified data will be made available to researchers upon request, subject to approval by the relevant institutional ethics committee and the implementation of appropriate data protection safeguards. Requests to access the datasets should be directed to nouraseada@gmail.com.
