# Supplementary material for: Exploring the factors associated with diabetes and hypertension complications among refugees living in Egypt
Source: Front Public Health. 2026 Sep 3;14:1818580. doi: 10.3389/fpubh.2026.1818580 (PMC13581648; doi:10.3389/fpubh.2026.1818580)
Supplement: Supplementary file 1 [file Table_1.DOCX]

# **Supplementary Material**

This file contains all supplementary material for the manuscript, comprising the questionnaire tools (Section 1), exploratory bivariate analyses (Section 2, Supplementary Tables S1–S4), and supporting analyses for the ordinal regression models (Section 3, Supplementary Tables S5–S9).

## **1. Questionnaire tools**

*An Arabic version of the forms below is available upon request. Three versions of the questionnaire were administered according to the participant's diagnosis: a combined form for participants with both hypertension and type 2 diabetes, a hypertension-only form, and a type 2 diabetes-only form. Items common to all three forms are identical in wording; the forms differ only in the disease-specific management and complication items.*

### **1.1 Questionnaire tool — combined hypertension and type 2 diabetes**

Age:         Weight (measured):

Gender:        Height (measured):

Nationality:       Area:

How long have you been in Egypt:  Phone number:

| **No.** | **Question** | **Coding categories** |
| --- | --- | --- |
| **General information** | | |
| 1 | What is your educational level? | None / Elementary / High school / Vocational training / University |
| 2 | Are you currently working? | Yes / No / Not regular working |
| 3 | What is your occupation? | Free text |
| **Disease management** | | |
| 4 | What chronic disease condition are you being treated for? | Hypertension / Diabetes / Both |
| 5 | How old were you when you were first told by a doctor or health professional that you had high blood pressure? | — |
| 6 | To lower your hypertension or high blood pressure, are you now taking prescribed medication? | Yes / No |
| 7 | How often do you measure blood pressure? | Weekly / Monthly / Occasionally or when I feel I need to / I don't measure |
| 8 | Do you have relatives who have the same condition? (hypertension) | Yes / No / I don't know |
| 9 | How old were you when you were first told by a doctor or health professional that you had diabetes? | — |
| 10 | Are you taking any prescribed medications for your diabetes? | Yes / No |
| 11 | What type of diabetic medications are you taking? | Insulin injections and pills / Pills only / Insulin only / None |
| 12 | How often do you measure blood sugar? | Daily / Weekly / Monthly / When I feel I need to |
| 13 | Do you have relatives who have the same condition? (diabetes) | Yes / No / I don't know |
| **Self-management and complications** | | |
| 14 | Have you noticed any blisters, cuts, or swelling in your feet in the last 2 weeks? | Yes / No / Don't know |
| 15 | Are you in the habit of checking your feet for these signs? | Yes, all the time / Yes, from time to time / Rarely / No / Not sure |
| 16 | Do you usually have suitable size footwear around your feet indoors and outdoors? | Yes, all the time / From time to time / Rarely / No / Not sure |
| 17 | Have you experienced diabetic foot complications (infection, gangrene, amputation) in the last year? | Yes / No / I don't know |
| 18 | Have you experienced diabetic eye complications (decreased vision, eye injections) during the last year? | Yes / No / I don't know |
| 19 | Has the doctor told you that your renal function has been affected? | Yes / No / I don't know |
| 20 | Have you experienced a heart attack or myocardial infarction or stroke (hospital admission)? | Yes / No / I don't know |
| 21 | In case of complications, have your medications been changed? | Changed partially / Changed totally / Change in doses / Nothing |
| **Lifestyle** | | |
| 22 | Do you currently smoke tobacco on a daily basis, less than daily, or not at all? | Daily / Less than daily / Not at all / Don't know |
| 23 | How many years ago did you first start smoking tobacco daily? | — |
| 24 | How many times do you eat fast food / food not prepared at home? | 4 times or more / 3 times / 2 times / ≤1 |
| 25 | How many times per week do you eat meat or chicken? | 4 times or more / 3 times / 2 times / ≤1 |
| 26 | How often do you eat fish per week? | 4 times or more / 3 times / 2 times / ≤1 |
| 27 | On a typical day, how much fruit would you eat? (one serving = 2 pieces of small fruit) | 2 or more / 1–2 / ≤1 / Almost none |
| 28 | How many servings of vegetables would you eat on a typical day? (½ cup cooked vegetables, 1 cup salad vegetables; e.g. tomatoes, carrots, cucumber, zucchini, green beans) | 2 or more / 1–2 / ≤1 / Almost none |
| 29 | How much rice, pasta, or bread would you eat on a typical day? (small cups / loaf) | 4 or more / 3 / 2 / ≤1 |
| 30 | What oil or fat ingredient do you use to cook your meals? | Vegetable oil / Natural butter / Vegetable margarine / Other / Mixture |
| 31 | How many times in the last 7 days have you engaged in physical activity for 30 minutes? (walking, using the stairs) | 4 or more / 3 / 2 / ≤1 |
| **Accessibility to healthcare services** | | |
| 32 | Has the healthcare professional explained to you the treatment plan and complications? | Yes / No |
| 33 | Are you taking your medications regularly? | Yes / No |
| 34 | Are you taking the exact medication types and dosage prescribed by your physician? | Yes (skip to Q36) / No |
| 35 | If no, what are your reasons? | Financial / Unavailability of medications / Too many medications / Unable to organise dosage and timing |
| 36 | During the last 3 months, did you need to receive care or treatment in a health facility? | Yes / No (skip to Q40) / Don't know |
| 37 | Were you able to access care? | Yes / No (skip to Q39) |
| 38 | If yes, where did you receive care? | Private clinic or hospital / Public hospital / NGO or charity clinic / Other |
| 39 | If no, what were the barriers? | Health centre refused care / Could not afford user fees / Too far or transportation issues / Could not get time off work or caring for children / Did not know where to go / Did not like the health services or staff / Language barrier / Lack of identity documents / Other / Don't know |
| 40 | Considering all your health conditions, do you think that your condition is under control with medications? | Yes, totally / Relatively / No / Not sure |
| **Social support (MOS-SS, tangible support subscale)** | | |
| 41 | Do you have someone who can help you when you are confined to bed? | None of the time / A little of the time / Some of the time / Most of the time / All of the time |
| 42 | Do you have someone to take you to the doctor if needed? | None of the time / A little of the time / Some of the time / Most of the time / All of the time |
| 43 | Do you have someone to prepare your meals if you are unable to do it yourself? | None of the time / A little of the time / Some of the time / Most of the time / All of the time |
| 44 | Do you have someone to help with daily chores if you were sick? | None of the time / A little of the time / Some of the time / Most of the time / All of the time |

### **1.2 Questionnaire tool — hypertension only**

Age:         Weight (measured):

Gender:        Height (measured):

Nationality:       Area:

How long have you been in Egypt:  Phone number:

| **No.** | **Question** | **Coding categories** |
| --- | --- | --- |
| **General information** | | |
| 1 | What is your educational level? | None / Elementary / High school / Vocational training / University |
| 2 | Are you currently working? | Yes / No / Not regular working |
| 3 | What is your occupation? | Free text |
| **Disease management** | | |
| 4 | What chronic disease condition are you being treated for? | Hypertension / Diabetes / Both |
| 5 | How old were you when you were first told by a doctor or health professional that you had high blood pressure? | — |
| 6 | To lower your hypertension or high blood pressure, are you now taking prescribed medication? | Yes / No |
| 7 | How often do you measure blood pressure? | Weekly / Monthly / Occasionally or when I feel I need to / I don't measure |
| 8 | Do you have relatives who have the same condition? (hypertension) | Yes / No / I don't know |
| **Self-management and complications** | | |
| 9 | Has the doctor told you that your renal function has been affected? | Yes / No / I don't know |
| 10 | Have you experienced a heart attack or myocardial infarction or stroke (hospital admission)? | Yes / No / I don't know |
| 11 | In case of complications, have your medications been changed? | Changed partially / Changed totally / Change in doses / Nothing |
| **Lifestyle** | | |
| 12 | Do you currently smoke tobacco on a daily basis, less than daily, or not at all? | Daily / Less than daily / Not at all / Don't know |
| 13 | How many years ago did you first start smoking tobacco daily? | — |
| 14 | How many times do you eat fast food / food not prepared at home? | 4 times or more / 3 times / 2 times / ≤1 |
| 15 | How many times per week do you eat meat or chicken? | 4 times or more / 3 times / 2 times / ≤1 |
| 16 | How often do you eat fish per week? | 4 times or more / 3 times / 2 times / ≤1 |
| 17 | On a typical day, how much fruit would you eat? (one serving = 2 pieces of small fruit) | 2 or more / 1–2 / ≤1 / Almost none |
| 18 | How many servings of vegetables would you eat on a typical day? (½ cup cooked vegetables, 1 cup salad vegetables; e.g. tomatoes, carrots, cucumber, zucchini, green beans) | 2 or more / 1–2 / ≤1 / Almost none |
| 19 | How much rice, pasta, or bread would you eat on a typical day? (small cups / loaf) | 4 or more / 3 / 2 / ≤1 |
| 20 | What oil or fat ingredient do you use to cook your meals? | Vegetable oil / Natural butter / Vegetable margarine / Other / Mixture |
| 21 | How many times in the last 7 days have you engaged in physical activity for 30 minutes? (walking, using the stairs) | 4 or more / 3 / 2 / ≤1 |
| **Accessibility to healthcare services** | | |
| 22 | Has the healthcare professional explained to you the treatment plan and complications? | Yes / No |
| 23 | Are you taking your medications regularly? | Yes / No |
| 24 | Are you taking the exact medication types and dosage prescribed by your physician? | Yes (skip to Q26) / No |
| 25 | If no, what are your reasons? | Financial / Unavailability of medications / Too many medications / Unable to organise dosage and timing |
| 26 | During the last 3 months, did you need to receive care or treatment in a health facility? | Yes / No (skip to Q30) / Don't know |
| 27 | Were you able to access care? | Yes / No (skip to Q29) |
| 28 | If yes, where did you receive care? | Private clinic or hospital / Public hospital / NGO or charity clinic / Other |
| 29 | If no, what were the barriers? | Health centre refused care / Could not afford user fees / Too far or transportation issues / Could not get time off work or caring for children / Did not know where to go / Did not like the health services or staff / Language barrier / Lack of identity documents / Other / Don't know |
| 30 | Considering all your health conditions, do you think that your condition is under control with medications? | Yes, totally / Relatively / No / Not sure |
| **Social support (MOS-SS, tangible support subscale)** | | |
| 31 | Do you have someone who can help you when you are confined to bed? | None of the time / A little of the time / Some of the time / Most of the time / All of the time |
| 32 | Do you have someone to take you to the doctor if needed? | None of the time / A little of the time / Some of the time / Most of the time / All of the time |
| 33 | Do you have someone to prepare your meals if you are unable to do it yourself? | None of the time / A little of the time / Some of the time / Most of the time / All of the time |
| 34 | Do you have someone to help with daily chores if you were sick? | None of the time / A little of the time / Some of the time / Most of the time / All of the time |

### **1.3 Questionnaire tool — type 2 diabetes only**

Age:         Weight (measured):

Gender:        Height (measured):

Nationality:       Area:

How long have you been in Egypt:  Phone number:

| **No.** | **Question** | **Coding categories** |
| --- | --- | --- |
| **General information** | | |
| 1 | What is your educational level? | None / Elementary / High school / Vocational training / University |
| 2 | Are you currently working? | Yes / No / Not regular working |
| 3 | What is your occupation? | Free text |
| **Disease management** | | |
| 4 | What chronic disease condition are you being treated for? | Hypertension / Diabetes / Both |
| 5 | How old were you when you were first told by a doctor or health professional that you had diabetes? | — |
| 6 | Are you taking any prescribed medications for your diabetes? | Yes / No |
| 7 | What type of diabetic medications are you taking? | Insulin injections and pills / Pills only / Insulin only / None |
| 8 | How often do you measure blood sugar? | Daily / Weekly / Monthly / When I feel I need to |
| 9 | Do you have relatives who have the same condition? (diabetes) | Yes / No / I don't know |
| **Self-management and complications** | | |
| 10 | Have you noticed any blisters, cuts, or swelling in your feet in the last 2 weeks? | Yes / No / Don't know |
| 11 | Are you in the habit of checking your feet for these signs? | Yes, all the time / Yes, from time to time / Rarely / No / Not sure |
| 12 | Do you usually have suitable size footwear around your feet indoors and outdoors? | Yes, all the time / From time to time / Rarely / No / Not sure |
| 13 | Have you experienced diabetic foot complications (infection, gangrene, amputation) in the last year? | Yes / No / I don't know |
| 14 | Have you experienced diabetic eye complications (decreased vision, eye injections) during the last year? | Yes / No / I don't know |
| 15 | Has the doctor told you that your renal function has been affected? | Yes / No / I don't know |
| 16 | Have you experienced a heart attack or myocardial infarction or stroke (hospital admission)? | Yes / No / I don't know |
| 17 | In case of complications, have your medications been changed? | Changed partially / Changed totally / Change in doses / Nothing |
| **Lifestyle** | | |
| 18 | Do you currently smoke tobacco on a daily basis, less than daily, or not at all? | Daily / Less than daily / Not at all / Don't know |
| 19 | How many years ago did you first start smoking tobacco daily? | — |
| 20 | How many times do you eat fast food / food not prepared at home? | 4 times or more / 3 times / 2 times / ≤1 |
| 21 | How many times per week do you eat meat or chicken? | 4 times or more / 3 times / 2 times / ≤1 |
| 22 | How often do you eat fish per week? | 4 times or more / 3 times / 2 times / ≤1 |
| 23 | On a typical day, how much fruit would you eat? (one serving = 2 pieces of small fruit) | 2 or more / 1–2 / ≤1 / Almost none |
| 24 | How many servings of vegetables would you eat on a typical day? (½ cup cooked vegetables, 1 cup salad vegetables; e.g. tomatoes, carrots, cucumber, zucchini, green beans) | 2 or more / 1–2 / ≤1 / Almost none |
| 25 | How much rice, pasta, or bread would you eat on a typical day? (small cups / loaf) | 4 or more / 3 / 2 / ≤1 |
| 26 | What oil or fat ingredient do you use to cook your meals? | Vegetable oil / Natural butter / Vegetable margarine / Other / Mixture |
| 27 | How many times in the last 7 days have you engaged in physical activity for 30 minutes? (walking, using the stairs) | 4 or more / 3 / 2 / ≤1 |
| **Accessibility to healthcare services** | | |
| 28 | Has the healthcare professional explained to you the treatment plan and complications? | Yes / No |
| 29 | Are you taking your medications regularly? | Yes / No |
| 30 | Are you taking the exact medication types and dosage prescribed by your physician? | Yes (skip to Q32) / No |
| 31 | If no, what are your reasons? | Financial / Unavailability of medications / Too many medications / Unable to organise dosage and timing |
| 32 | During the last 3 months, did you need to receive care or treatment in a health facility? | Yes / No (skip to Q36) / Don't know |
| 33 | Were you able to access care? | Yes / No (skip to Q35) |
| 34 | If yes, where did you receive care? | Private clinic or hospital / Public hospital / NGO or charity clinic / Other |
| 35 | If no, what were the barriers? | Health centre refused care / Could not afford user fees / Too far or transportation issues / Could not get time off work or caring for children / Did not know where to go / Did not like the health services or staff / Language barrier / Lack of identity documents / Other / Don't know |
| 36 | Considering all your health conditions, do you think that your condition is under control with medications? | Yes, totally / Relatively / No / Not sure |
| **Social support (MOS-SS, tangible support subscale)** | | |
| 37 | Do you have someone who can help you when you are confined to bed? | None of the time / A little of the time / Some of the time / Most of the time / All of the time |
| 38 | Do you have someone to take you to the doctor if needed? | None of the time / A little of the time / Some of the time / Most of the time / All of the time |
| 39 | Do you have someone to prepare your meals if you are unable to do it yourself? | None of the time / A little of the time / Some of the time / Most of the time / All of the time |
| 40 | Do you have someone to help with daily chores if you were sick? | None of the time / A little of the time / Some of the time / Most of the time / All of the time |

## **2. Bivariate analyses**

Supplementary Tables S1 to S4 present exploratory bivariate analyses. Supplementary Table S1 reports categorical disease, complication, treatment, and healthcare-access outcomes across five sociodemographic characteristics in a single table, so that comparisons across characteristics can be read directly from one place. Educational level is reported separately in Supplementary Table S2 because it has three categories. Supplementary Table S3 reports continuous variables across the same characteristics, and Supplementary Table S4 gives the correlation matrix.

These analyses were exploratory and were not corrected for multiple comparisons; they describe the distribution of characteristics within this population rather than supporting confirmatory inference.

**Supplementary Table S1. Disease state, complications, treatment, and healthcare access by sociodemographic characteristics**

| **Variable** | **Response** | **Age group** | | | **Gender** | | | **Nationality** | | | **Duration in Egypt** | | | **Employment** | | |
| --- | --- | --- | --- | --- | --- | --- | --- | --- | --- | --- | --- | --- | --- | --- | --- | --- |
|  |  | **<50** | **≥50** | **χ² (p)** | **Female** | **Male** | **χ² (p)** | **Syrian** | **Sudanese** | **χ² (p)** | **<4 y** | **≥4 y** | **χ² (p)** | **Not working** | **Working** | **χ² (p)** |
| Hypertension (HTN) | Yes | 66 (70.2) | 105 (71.4) | 0.04 (.839) | 132 (72.9) | 42 (64.6) | 1.60 (.206) | 33 (86.8) | 139 (67.5) | **5.79 (.016)** | 130 (71.4) | 39 (69.6) | 0.07 (.797) | 132 (72.9) | 34 (59.6) | 3.62 (.057) |
|  | No | 28 (29.8) | 42 (28.6) |  | 49 (27.1) | 23 (35.4) |  | 5 (13.2) | 67 (32.5) |  | 52 (28.6) | 17 (30.4) |  | 49 (27.1) | 23 (40.4) |  |
| Type 2 diabetes (T2D) | Yes | 48 (51.1) | 99 (67.3) | **6.39 (.011)** | 105 (58.0) | 47 (72.3) | **4.14 (.042)** | 21 (55.3) | 129 (62.6) | 0.73 (.392) | 109 (59.9) | 36 (64.3) | 0.35 (.556) | 119 (65.7) | 30 (52.6) | 3.19 (.074) |
|  | No | 46 (48.9) | 48 (32.7) |  | 76 (42.0) | 18 (27.7) |  | 17 (44.7) | 77 (37.4) |  | 73 (40.1) | 20 (35.7) |  | 62 (34.3) | 27 (47.4) |  |
| Both HTN and T2D | Yes | 20 (21.3) | 57 (38.8) | **8.08 (.004)** | 56 (30.9) | 24 (36.9) | 0.78 (.377) | 16 (42.1) | 62 (30.1) | 2.13 (.145) | 57 (31.3) | 19 (33.9) | 0.13 (.714) | 70 (38.7) | 7 (12.3) | **13.80 (<.001)** |
|  | No | 74 (78.7) | 90 (61.2) |  | 125 (69.1) | 41 (63.1) |  | 22 (57.9) | 144 (69.9) |  | 125 (68.7) | 37 (66.1) |  | 111 (61.3) | 50 (87.7) |  |
| BMI category | Normal | 16 (18.8) | 35 (25.2) | 2.29 (.318) | 30 (17.6) | 22 (37.3) | **14.01 (.001)** | 5 (13.9) | 47 (23.6) | 2.66 (.265) | 42 (24.9) | 8 (15.4) | 2.91 (.234) | 35 (20.5) | 15 (29.4) | 1.80 (.406) |
|  | Overweight | 29 (34.1) | 52 (37.4) |  | 58 (34.1) | 23 (39.0) |  | 12 (33.3) | 67 (35.1) |  | 62 (36.7) | 18 (34.6) |  | 60 (35.1) | 16 (31.4) |  |
|  | Obese | 40 (47.1) | 52 (37.4) |  | 82 (48.2) | 14 (23.7) |  | 19 (52.8) | 77 (40.3) |  | 65 (38.5) | 26 (50.0) |  | 76 (44.4) | 20 (39.2) |  |
| Diabetic foot complications in the past year | Yes | 5 (5.3) | 11 (7.5) | 0.43 (.510) | 8 (7.9) | 9 (19.6) | **4.19 (.041)** | 3 (15.8) | 14 (11.1) | .734† | 10 (5.5) | 6 (10.7) | .219† | 12 (10.4) | 5 (17.2) | .563† |
|  | No | 89 (94.7) | 136 (92.5) |  | 93 (92.1) | 37 (80.4) |  | 16 (84.2) | 112 (88.9) |  | 172 (94.5) | 50 (89.3) |  | 103 (89.6) | 24 (82.8) |  |
| Diabetic eye complications in the past year | Yes | 31 (33.0) | 54 (36.7) | 0.35 (.552) | 56 (54.9) | 30 (65.2) | 1.39 (.239) | 11 (55.0) | 73 (57.9) | 0.61 (.805) | 58 (31.9) | 25 (44.6) | 3.08 (.079) | 62 (53.4) | 23 (79.3) | **6.40 (.011)** |
|  | No | 63 (67.0) | 93 (63.3) |  | 46 (45.1) | 16 (34.8) |  | 9 (45.0) | 53 (42.1) |  | 124 (68.1) | 31 (55.4) |  | 54 (46.6) | 6 (20.7) |  |
| Renal function affected (doctor-reported) | Yes | 11 (11.7) | 23 (15.6) | 0.74 (.391) | 25 (14.0) | 9 (14.8) | 0.02 (.891) | 13 (35.1) | 21 (10.5) | **15.42 (<.001)** | 14 (7.7) | 19 (33.9) | **24.68 (<.001)** | 23 (13.1) | 10 (17.5) | 0.68 (.409) |
|  | No | 83 (88.3) | 124 (84.4) |  | 153 (86.0) | 52 (85.2) |  | 24 (64.9) | 179 (89.5) |  | 168 (92.3) | 37 (66.1) |  | 152 (86.9) | 47 (82.5) |  |
| Heart attack or stroke | Yes | 6 (6.4) | 30 (20.4) | **8.88 (.003)** | 24 (13.6) | 12 (18.8) | 0.96 (.327) | 14 (37.8) | 22 (10.9) | **17.60 (<.001)** | 18 (9.9) | 18 (32.1) | **16.52 (<.001)** | 30 (17.1) | 5 (8.8) | 2.35 (.125) |
|  | No | 88 (93.6) | 117 (79.6) |  | 152 (86.4) | 52 (81.3) |  | 23 (62.2) | 179 (89.1) |  | 164 (90.1) | 38 (67.9) |  | 145 (82.9) | 52 (91.2) |  |
| Medications changed due to complications | Yes | 52 (63.4) | 74 (52.9) | 2.35 (.125) | 93 (56.7) | 35 (56.5) | 0.00 (.972) | 28 (77.8) | 99 (52.7) | **7.76 (.005)** | 87 (53.0) | 37 (68.5) | **3.96 (.046)** | 86 (51.8) | 39 (73.6) | **7.78 (.005)** |
|  | No | 30 (36.6) | 66 (47.1) |  | 71 (43.3) | 27 (43.5) |  | 8 (22.2) | 89 (47.3) |  | 77 (47.0) | 17 (31.5) |  | 80 (48.2) | 14 (26.4) |  |
| Taking medications regularly | Yes | 62 (77.5) | 114 (89.1) | **5.06 (.025)** | 128 (84.2) | 53 (86.9) | 0.24 (.621) | 33 (91.7) | 146 (83.4) | 1.58 (.209) | 131 (84.0) | 44 (88.0) | 0.48 (.488) | 139 (87.4) | 36 (76.6) | 3.33 (.068) |
|  | No | 18 (22.5) | 14 (10.9) |  | 24 (15.8) | 8 (13.1) |  | 3 (8.3) | 29 (16.6) |  | 25 (16.0) | 6 (12.0) |  | 20 (12.6) | 11 (23.4) |  |
| Taking exact medication types and dosage prescribed | Yes | 51 (57.3) | 88 (62.4) | 0.60 (.440) | 101 (58.4) | 41 (66.1) | 0.24 (.680) | 13 (34.2) | 128 (65.6) | **13.15 (<.001)** | 116 (67.4) | 24 (43.6) | **9.99 (.002)** | 107 (61.5) | 31 (57.4) | 0.29 (.591) |
|  | No | 38 (42.7) | 53 (37.6) |  | 72 (41.6) | 21 (33.9) |  | 25 (65.8) | 67 (34.4) |  | 56 (32.6) | 31 (56.4) |  | 67 (38.5) | 23 (42.6) |  |
| Needed care in the past 3 months | Yes | 48 (51.1) | 74 (51.4) | 0.00 (.961) | 95 (53.4) | 31 (47.7) | 0.68 (.409) | 19 (50.0) | 105 (51.7) | 0.38 (.845) | 89 (49.4) | 34 (61.8) | 2.59 (.108) | 90 (50.0) | 30 (53.6) | 0.22 (.641) |
|  | No | 46 (48.9) | 70 (48.6) |  | 83 (46.6) | 34 (52.3) |  | 19 (50.0) | 98 (48.3) |  | 91 (50.6) | 21 (38.2) |  | 90 (50.0) | 26 (46.4) |  |
| Able to access care when needed | Yes | 41 (63.1) | 71 (67.6) | 0.37 (.544) | 87 (66.4) | 29 (67.4) | 0.02 (.901) | 13 (54.2) | 101 (68.2) | 1.83 (.176) | 84 (67.7) | 31 (70.5) | 0.11 (.739) | 84 (66.1) | 27 (65.9) | 0.00 (.973) |
|  | No | 24 (36.9) | 34 (32.4) |  | 44 (33.6) | 14 (32.6) |  | 11 (45.8) | 47 (31.8) |  | 40 (32.3) | 13 (29.5) |  | 43 (33.9) | 14 (34.1) |  |
| Condition under control with medications | Yes, totally | 21 (22.6) | 63 (43.4) | **15.52 (<.001)** | 65 (36.1) | 23 (36.5) | 2.82 (.245) | 9 (23.7) | 78 (38.4) | **7.14 (.028)** | 67 (37.4) | 18 (32.1) | 3.98 (.137) | 69 (38.8) | 16 (28.1) | 3.28 (.194) |
|  | Relatively | 46 (49.5) | 65 (44.8) |  | 79 (43.9) | 33 (52.4) |  | 17 (44.7) | 95 (46.8) |  | 85 (47.5) | 23 (41.1) |  | 81 (45.5) | 27 (47.4) |  |
|  | No | 26 (28.0) | 17 (11.7) |  | 36 (20.0) | 7 (11.1) |  | 12 (31.6) | 30 (14.8) |  | 27 (15.1) | 15 (26.8) |  | 28 (15.7) | 14 (24.6) |  |
|  | | | | | | | | | | | | | | | | |

*n (%), number and column percentage; χ², chi-square statistic; HTN, hypertension; T2D, type 2 diabetes; BMI, body mass index. Bold χ² (p) values indicate statistical significance (p < .05).*

*Denominators vary by row and reflect valid responses only. Diabetic foot and eye complication items were administered only to participants with T2D; the item on ability to access care only to participants reporting a need for care in the preceding three months; medication items only to participants reporting current medication use. Age and duration of stay were dichotomised at the sample mean for these exploratory comparisons only; both were retained as continuous variables in the regression models.*

*† Fisher's exact test used in place of the chi-square approximation because at least one expected cell count was below 5; no χ² statistic is reported for these comparisons.*

**Supplementary Table S2. Disease state, complications, treatment, and healthcare access by educational level**

| **Variable** | **Response** | **Up to elementary N (%)** | **High school N (%)** | **Vocational or university N (%)** | **χ²** | **p-value** |
| --- | --- | --- | --- | --- | --- | --- |
| Hypertension (HTN) | Yes | 41 (73.2%) | 61 (68.5%) | 68 (70.1%) | 0.36 | .835 |
|  | No | 15 (26.8%) | 28 (31.5%) | 29 (29.9%) |  |  |
| Type 2 diabetes (T2D) | Yes | 37 (66.1%) | 60 (67.4%) | 52 (53.6%) | 4.36 | .113 |
|  | No | 19 (33.9%) | 29 (32.6%) | 45 (46.4%) |  |  |
| Both HTN and T2D | Yes | 22 (39.3%) | 32 (36.0%) | 23 (23.7%) | 5.08 | .079 |
|  | No | 34 (60.7%) | 57 (64.0%) | 74 (76.3%) |  |  |
| BMI category | Normal | 13 (24.5%) | 19 (22.6%) | 19 (21.6%) | 1.41 | .843 |
|  | Overweight | 16 (30.2%) | 29 (34.5%) | 35 (39.8%) |  |  |
|  | Obese | 24 (45.3%) | 36 (42.9%) | 34 (38.6%) |  |  |
| Diabetic foot complications in the past year | Yes | 7 (12.5%) | 5 (5.6%) | 5 (5.2%) | — | .243† |
|  | No | 49 (87.5%) | 84 (94.4%) | 92 (94.8%) |  |  |
| Diabetic eye complications in the past year | Yes | 19 (33.9%) | 38 (42.7%) | 27 (27.8%) | 4.54 | .103 |
|  | No | 37 (66.1%) | 51 (57.3%) | 70 (72.2%) |  |  |
| Renal function affected (doctor-reported) | Yes | 9 (16.1%) | 17 (19.1%) | 7 (7.2%) | 5.93 | .051 |
|  | No | 47 (83.9%) | 72 (80.9%) | 90 (92.8%) |  |  |
| Heart attack or stroke | Yes | 9 (16.1%) | 16 (18.0%) | 10 (10.3%) | 2.36 | .308 |
|  | No | 47 (83.9%) | 73 (82.0%) | 87 (89.7%) |  |  |
| Medications changed due to complications | Yes | 29 (52.7%) | 48 (60.0%) | 49 (56.3%) | 0.71 | .700 |
|  | No | 26 (47.3%) | 32 (40.0%) | 38 (43.7%) |  |  |
| Taking medications regularly | Yes | 41 (83.7%) | 70 (89.7%) | 66 (80.5%) | 2.69 | .260 |
|  | No | 8 (16.3%) | 8 (10.3%) | 16 (19.5%) |  |  |
| Taking exact medication types and dosage prescribed | Yes | 27 (50.9%) | 49 (57.0%) | 64 (69.6%) | 5.64 | .060 |
|  | No | 26 (49.1%) | 37 (43.0%) | 28 (30.4%) |  |  |
| Needed care in the past 3 months | Yes | 30 (55.6%) | 51 (58.0%) | 43 (44.3%) | 3.81 | .149 |
|  | No | 24 (44.4%) | 37 (42.0%) | 54 (55.7%) |  |  |
| Able to access care when needed | Yes | 24 (66.7%) | 44 (63.8%) | 46 (68.7%) | 0.37 | .833 |
|  | No | 12 (33.3%) | 25 (36.2%) | 21 (31.3%) |  |  |
| Condition under control with medications | Yes, totally | 28 (50.9%) | 27 (31.0%) | 30 (30.9%) | 7.92 | .095 |
|  | Relatively | 18 (32.7%) | 45 (51.7%) | 48 (49.5%) |  |  |
|  | No | 9 (16.4%) | 15 (17.2%) | 19 (19.6%) |  |  |
|  | | | | | | |

*N (%), number and column percentage; χ², chi-square statistic; HTN, hypertension; T2D, type 2 diabetes; BMI, body mass index. Bold p-values indicate statistical significance (p < .05).*

*Denominators vary by row and reflect valid responses only. Diabetic foot and eye complication items were administered only to participants with T2D; the item on ability to access care only to participants reporting a need for care in the preceding three months; medication items only to participants reporting current medication use.*

*† Fisher–Freeman–Halton exact test used in place of the chi-square approximation because at least one expected cell count was below 5; no χ² statistic is reported for this comparison.*

**Supplementary Table S3. Age, anthropometric, and tangible social support measures by gender, nationality, and employment status**

| **Variable** | **Comparison** | **Group 1 Mean (SD)** | **Group 2 Mean (SD)** | **Mean difference [95% CI]** | **p-value** |
| --- | --- | --- | --- | --- | --- |
| Age (years) | Female vs male | 50.41 (10.40) | 60.44 (10.49) | −10.03 [−13.02, −7.03] | **<.001** |
|  | Syrian vs Sudanese | 56.74 (10.10) | 52.36 (11.47) | +4.37 [0.45, 8.30] | **.029** |
|  | Not working vs working | 54.73 (11.30) | 48.14 (10.44) | +6.59 [3.37, 9.82] | **<.001** |
| Years in Egypt | Female vs male | 3.81 (4.57) | 2.94 (3.65) | +0.87 [−0.39, 2.13] | .174 |
|  | Syrian vs Sudanese | 11.17 (5.80) | 2.29 (2.22) | +8.88 [6.87, 10.89] | **<.001** |
|  | Not working vs working | 3.70 (4.66) | 3.54 (3.54) | +0.16 [−1.01, 1.34] | .783 |
| BMI (kg/m²) | Female vs male | 30.22 (6.02) | 27.38 (4.91) | +2.84 [1.13, 4.54] | **.001** |
|  | Syrian vs Sudanese | 30.16 (5.41) | 29.38 (6.00) | +0.77 [−1.34, 2.89] | .472 |
|  | Not working vs working | 29.84 (5.87) | 28.93 (5.96) | +0.92 [−0.95, 2.78] | .332 |
| Years since starting daily smoking | Female vs male | 18.67 (15.71) | 28.92 (14.76) | −10.25 [−26.21, 5.71] | .192 |
|  | Syrian vs Sudanese | 31.43 (14.92) | 21.73 (15.20) | +9.70 [−5.77, 25.17] | .202 |
|  | Not working vs working | 24.92 (17.33) | 26.67 (12.11) | −1.75 [−16.85, 13.35] | .807 |
| Age at diagnosis of HTN (years) | Female vs male | 40.42 (9.86) | 49.03 (12.46) | −8.61 [−13.29, −3.93] | **.001** |
|  | Syrian vs Sudanese | 45.07 (10.83) | 41.65 (11.14) | +3.42 [−1.05, 7.89] | .133 |
|  | Not working vs working | 42.98 (11.36) | 40.34 (10.24) | +2.64 [−1.56, 6.84] | .213 |
| Age at diagnosis of T2D (years) | Female vs male | 41.08 (10.49) | 48.33 (12.97) | −7.25 [−11.36, −3.15] | **.001** |
|  | Syrian vs Sudanese | 46.00 (9.41) | 42.76 (12.12) | +3.24 [−2.40, 8.87] | .258 |
|  | Not working vs working | 43.89 (11.99) | 40.72 (10.45) | +3.17 [−1.35, 7.69] | .165 |
| Social support mean score | Female vs male | 3.17 (1.23) | 3.71 (1.13) | −0.54 [−0.89, −0.19] | **.003** |
|  | Syrian vs Sudanese | 2.85 (1.22) | 3.39 (1.22) | −0.54 [−0.99, −0.09] | **.018** |
|  | Not working vs working | 3.36 (1.25) | 3.16 (1.19) | +0.20 [−0.17, 0.57] | .289 |
|  | | | | | |

*SD, standard deviation; CI, confidence interval; HTN, hypertension; T2D, type 2 diabetes; BMI, body mass index. Bold p-values indicate statistical significance (p < .05).*

*Group 1 and Group 2 correspond to the first and second category named in the Comparison column. All comparisons by independent-samples t-test; a positive mean difference indicates a higher value in Group 1. Denominators vary by variable and reflect valid responses only; age at diagnosis of HTN and of T2D were asked only of participants with the respective diagnosis, and years since starting daily smoking only of participants reporting daily smoking.*

**Supplementary Table S4. Correlation matrix of continuous study variables**

Age was positively correlated with age at diagnosis of both conditions and with social support, and inversely correlated with BMI. Social support was also inversely associated with BMI. Coefficients are reported in full below and are not repeated in the text.

| **Variable** | **1** | **2** | **3** | **4** | **5** | **6** | **7** | **M** | **SD** |
| --- | --- | --- | --- | --- | --- | --- | --- | --- | --- |
| 1. Age (years) | – |  |  |  |  |  |  | 53.07 | 11.31 |
| 2. Age at diagnosis of HTN (years) | **.693*** | – |  |  |  |  |  | 42.49 | 11.06 |
| 3. Age at diagnosis of T2D (years) | **.672*** | **.718*** | – |  |  |  |  | 43.24 | 11.72 |
| 4. Age at starting daily smoking (years) | .359 | .373 | −.374 | – |  |  |  | 25.50 | 15.43 |
| 5. Years in Egypt | .027 | −.005 | −.035 | .296 | – |  |  | 3.85 | 4.36 |
| 6. BMI (kg/m²) | **−.147*** | −.159 | −.005 | −.034 | .098 | – |  | 29.50 | 5.88 |
| 7. Social support sum score | **.288*** | .066 | **.262*** | −.206 | −.128 | **−.179*** | – | 13.24 | 4.90 |
|  | | | | | | | | | |

*M, mean; SD, standard deviation; HTN, hypertension; T2D, type 2 diabetes; BMI, body mass index. Values are Pearson correlation coefficients (r). * p < .05. Bold values indicate statistically significant correlations. Denominators vary by pair and reflect valid responses only.*

## **3. Supporting analyses for the ordinal regression models**

Supplementary Tables S5 to S8 document the derivation of the analytic samples for the two ordinal regression models and compare participants included in each model with those excluded because of missing data. Supplementary Table S9 reports multicollinearity diagnostics for both models.

**Supplementary Table S5. Missing data flow for the cardiac and renal complications regression model (full sample, N = 246)**

| **Stage** | **N remaining** | **N excluded** | **Reason** |
| --- | --- | --- | --- |
| Total study participants | 246 | — | *Full sample* |
| Excluded: missing on medication compliance | 208 | 38 | *Item non-response to “Are you taking your medications regularly?”, which determines the compliance classification* |
| Excluded: missing on remaining covariates | 169 | 39 | *Listwise deletion across BMI (n = 14), social support score (n = 15), years in Egypt (n = 8), access to care (n = 6), age (n = 5), and nationality (n = 2); some participants were missing on more than one variable* |
| **Final analytic sample** | **169** | **—** | ***Used in the ordinal logistic regression model*** |
|  | | | |

*N, number of participants; BMI, body mass index.*

**Supplementary Table S6. Comparison of participants included in and excluded from the cardiac and renal complications regression model (full sample, N = 246)**

| **Characteristic** | **Category** | **Included (n = 169)** | **Excluded (n = 77)** | **p-value** |
| --- | --- | --- | --- | --- |
| ***Gender (n = 246)*** | | | | |
|  | Female | 122 (74.4%) | 59 (76.6%) | .465 |
|  | Male | 47 (25.6%) | 18 (23.4%) |  |
| ***Nationality (n = 244)¹*** | | | | |
|  | Syrian | 28 (17.5%) | 10 (13.3%) | .520 |
|  | Sudanese | 141 (82.5%) | 65 (86.7%) |  |
| ***Social support score (n = 231)²*** | | | | |
|  | 1 – lowest | 13 (8.1%) | 5 (7.8%) | .785 |
|  | 2 | 39 (24.4%) | 18 (28.1%) |  |
|  | 3 | 44 (27.5%) | 16 (25.0%) |  |
|  | 4 – highest | 73 (45.6%) | 23 (35.9%) |  |
| ***Continuous variables*** | | | | |
| Years in Egypt | Mean (SD) | 3.57 (4.22) | 3.62 (4.71) | .933 |
| BMI (kg/m²) | Mean (SD) | 29.30 (5.76) | 30.01 (6.21) | .412 |
| Age (years) | Mean (SD) | 53.22 (12.03) | 52.74 (9.47) | .762 |
|  | | | | |

*N (%), number and percentage; SD, standard deviation; BMI, body mass index. Categorical variables compared by Pearson chi-square test; continuous variables by independent-samples t-test. Bold p-values indicate statistical significance (p < .05).*

*¹ Two participants with missing nationality data were excluded from this comparison (n = 244).*

*² Denominator reflects valid responses only; participants missing on this variable were excluded from this comparison.*

*No statistically significant differences were observed between included and excluded participants on any characteristic examined (all p > .05), supporting the assumption that missingness was largely unrelated to these measured characteristics.*

**Supplementary Table S7. Missing data flow for the diabetic foot and eye complications regression model (full sample, N = 246)**

| **Stage** | **N remaining** | **N excluded** | **Reason** |
| --- | --- | --- | --- |
| Total study participants | 246 | — | *Full sample* |
| Excluded: no T2D diagnosis | 152 | 94 | *Did not meet the diagnostic inclusion criterion for this model* |
| Excluded: missing on medication compliance | 135 | 17 | *Item non-response to “Are you taking your medications regularly?”, which determines the compliance classification* |
| Excluded: missing on remaining covariates | 110 | 25 | *Listwise deletion across social support score (n = 11), BMI (n = 9), years in Egypt (n = 7), access to care (n = 3), rice, pasta or bread intake (n = 2), and nationality (n = 2); some participants were missing on more than one variable* |
| **Final analytic sample** | **110** | **—** | ***Used in the ordinal logistic regression model*** |
|  | | | |

*N, number of participants; T2D, type 2 diabetes; BMI, body mass index.*

**Supplementary Table S8. Comparison of participants included in and excluded from the diabetic foot and eye complications regression model (T2D-eligible participants, n = 152)**

| **Characteristic** | **Category** | **Included (n = 110)** | **Excluded (n = 42)** | **p-value** |
| --- | --- | --- | --- | --- |
| ***Gender (n = 152)*** | | | | |
|  | Female | 74 (67.3%) | 31 (73.8%) | .436 |
|  | Male | 36 (32.7%) | 11 (26.2%) |  |
| ***Nationality (n = 150)¹*** | | | | |
|  | Syrian | 14 (12.7%) | 7 (17.5%) | .456 |
|  | Sudanese | 96 (87.3%) | 33 (82.5%) |  |
| ***Social support score (n = 141)²*** | | | | |
|  | 1 – lowest | 6 (5.5%) | 3 (9.7%) | .666 |
|  | 2 | 26 (23.6%) | 9 (29.0%) |  |
|  | 3 | 30 (27.3%) | 6 (19.4%) |  |
|  | 4 – highest | 48 (43.6%) | 13 (41.9%) |  |
| ***Healthcare access (n = 149)²*** | | | | |
|  | Did not need care | 51 (46.4%) | 19 (48.7%) | .949 |
|  | Sought but could not access | 10 (9.1%) | 3 (7.7%) |  |
|  | Accessed care | 49 (44.5%) | 17 (43.6%) |  |
| ***Medication compliance (n = 135)²*** | | | | |
|  | Not compliant | 13 (11.8%) | 2 (8.0%) | **.025** |
|  | Regular but not exact prescription | 27 (24.5%) | 13 (52.0%) |  |
|  | Exact prescription | 70 (63.6%) | 10 (40.0%) |  |
| ***Rice, pasta or bread intake, servings per day (n = 150)²*** | | | | |
|  | 4 or more | 20 (18.2%) | 5 (12.5%) | .871 |
|  | 3 | 27 (24.5%) | 11 (27.5%) |  |
|  | 2 | 37 (33.6%) | 14 (35.0%) |  |
|  | ≤1 | 26 (23.6%) | 10 (25.0%) |  |
| ***Continuous variables*** | | | | |
| Years in Egypt | Mean (SD) | 3.35 (3.70) | 3.46 (3.77) | .879 |
| BMI (kg/m²) | Mean (SD) | 29.39 (5.67) | 30.27 (5.90) | .443 |
|  | | | | |

*N (%), number and percentage; SD, standard deviation; T2D, type 2 diabetes; BMI, body mass index. Comparisons are restricted to the 152 participants meeting the T2D diagnostic inclusion criterion. Categorical variables compared by Pearson chi-square test; continuous variables by independent-samples t-test. Bold p-values indicate statistical significance (p < .05).*

*¹ Two participants with missing nationality data were excluded from this comparison (n = 150).*

*² Denominator reflects valid responses only; participants missing on this variable were excluded from this comparison.*

*Medication compliance was the only characteristic showing a statistically significant difference between included and excluded participants (p = .025): excluded participants were more likely to report taking medications regularly but not exactly as prescribed, and less likely to report taking the exact prescription. This is acknowledged in the manuscript as a potential source of complete-case bias in the interpretation of the compliance finding.*

**Supplementary Table S9. Variance inflation factors for predictors included in the final ordinal regression models**

| **Predictor** | **Diabetic foot and eye complications model VIF** | **Cardiac and renal complications model VIF** |
| --- | --- | --- |
| Gender | 1.10 | 1.33 |
| Nationality | 2.10 | 2.42 |
| Social support score | 1.06 | 1.19 |
| Healthcare access | 1.07 | 1.05 |
| Medication compliance | 1.02 | 1.04 |
| Years in Egypt | 2.11 | 2.31 |
| BMI (kg/m²) | 1.09 | 1.09 |
| Age (years) | — | 1.56 |
| Rice, pasta or bread intake (servings per day) | 1.03 | — |
|  | | |

*VIF, variance inflation factor; BMI, body mass index. VIF and tolerance statistics were derived from auxiliary linear regression models specified with the same predictor sets as the final ordinal regression models, because variance inflation diagnostics are not produced directly by the ordinal logistic regression procedure. All VIF values were below the conventional threshold of 5, indicating no problematic multicollinearity in either model. An em dash (—) indicates that the predictor was not included in that model.*
